# 4-Nitrophenol Efficient Photoreduction from Exfoliated and Protonated Phenyl-Doped Graphitic Carbon Nitride Nanosheets

**DOI:** 10.3390/polym13213752

**Published:** 2021-10-29

**Authors:** Stefania Porcu, Francesco Secci, Qader Abdulqader Abdullah, Pier Carlo Ricci

**Affiliations:** 1 Department of Physics, University of Cagliari, 09042 Monserrato, Italy; carlo.ricci@dsf.unica.it; 2 Department of Chemical and Geological Science, University of Cagliari, 09042 Monserrato, Italy; fsecci@unica.it; 3 Department of Physics, University of Sulaimani, Kirkuk Road, Sulaimani 46001, Kurdistan Region, Iraq; abdulqader.qader@univsul.edu.iq

**Keywords:** phenyl modified carbon nitride, photoreduction, 4-nitrophenol, photocatalysis, surface and bulk modification, functionalization, pollutants degradation, g-C_3_N_4_

## Abstract

The photoreduction of 4-nitrophenol to 4-aminophenol by means of protonated and exfoliated phenyl-doped carbon nitride is reported. Although carbon nitride-based materials have been recognized as efficient photocatalysts, the photoreduction of 4-nitrophenol to 4-aminophenol is not allowed because of the high recombination rate of the photogenerated electron–hole pairs. In this paper, we show the morphology effects on the photoactivity in phenyl-doped carbon nitride. Structural (TEM, XRD, Raman) and optical characterization (absorption, photoluminescence) of the protonated and exfoliated phenyl-doped carbon nitride (hereafter pePhCN) is reported. The increased photocatalytic efficiency, with respect to the bulk material, is underlined by the calculation of the kinetic constant of the photoreduction process (2.78 × 10^−1^ min^−1^ and 3.54 × 10^−3^ min^−1^) for pePhCN and bulk PhCN, respectively. Finally, the detailed mechanism of the photoreduction process of 4-nitrophenol to 4-aminophenol by modified phenyl carbon nitride is proposed.

## 1. Introduction

Materials research and development is boosted by a continuous demand for clean energy production, optoelectronic and electronic innovations, and in recent years, carbon nitride materials have attracted large interest from researchers [1,2,3]. These materials represent a class of 2D polymeric materials mainly composed of carbon and nitrogen, with high potential in the areas of sensing, imaging, and therapy due to their unique optical and electronic properties. 

Carbon nitride can be easily prepared at low cost from a variety of nitrogen-rich materials, where one of the biggest challenges that remains is the control of the valence and conduction band positions varying the nitrogen/carbon ratio [4].

Graphitic carbon nitride (g-C_3_N_4_) is the most studied material of this family for its mild band gap (2.7 eV), flexibility, chemical and thermal stability, with large potential in photocatalysis and in photonic applications [2,5]. Despite that in the field of photocatalysis the use of semiconductor is a promising pathway for the degradation of environmental pollutants [6,7], g-C_3_N_4_ does not represent a good standalone photocatalytic material for the high recombination rate of photogenerated electron–hole pairs. One possible strategy is the development of hybrid organic–inorganic materials, where the formation of charge-transfer complex at the interface between the organic donor (i.e., g-C_3_N_4_) and inorganic acceptor (TiO_2_/WO_3_) permits to increase the charge separation and, consequently, the photocatalytic activities [8,9,10,11].

The increased concentration of nitrogen oxides (NOx) in the atmosphere requires the development of new technologies for rapid and complete degradation of organic pollutants and semiconductor photocatalysis, one of the greenest and energy-saving strategies [12,13,14,15,16,17].

In this regard, the reduction of nitro aromatic compounds into amino aromatic compounds is one of the most studied processes. 4-nitrophenol (hereafter 4-NP), due to its high chemical stability and toxicity, has been classified by the EPA (United States Environmental Protection Agency) as one of the major pollutants and its reduction allows to obtain 4-aminophenol (hereafter 4-AP). Further, 4-AP is widely used in the manufacturing of many analgesics and antipyretics such as paracetamol, acetanilide, phenacetin [18,19,20]. 

During the photocatalytic process, the photoexcited electrons on the conduction band (CB) of photocatalysts reduce 4-NP to 4-AP, and the corresponding photogenerated holes on the valence band (VB) promote the photo-oxidation of organic compounds, including 4-NP. To prevent the oxidation and to improve the photocatalytic reduction of 4-NP the competitive oxidation reaction can be suppressed using hole scavengers such as NaBH_4_, Na_2_SO_3_ or hydrazine [21,22,23].

For an efficient photoreduction a good separation of photoexcited electron pairs is required, and the photogenerated electrons must rapidly react at the photocatalyst surface with the 4-NP.

In the case of carbon nitride materials, the catalytic performance is currently narrowed by the poor electrical transport, low density of reactive sites and high recombination of photoexcited electron–hole pairs [24]. 

Recent studies reported that controlling the morphology [25,26,27,28,29] and the surface properties of these catalysts induces significant enhancement in photoreduction. In particular, the protonation using different mineral acids (HCl, H_2_SO_4_, HNO_3_) is considered as a facile and effective method [30,31] to improve the solubility, dispersibility and electronic structure. Further, the exfoliation method of 2D structures, through the breaking of Van der Waals forces, can improve the absorption of the light [32,33,34,35,36,37,38,39]. This strategy has been used for many different applications such as water splitting for hydrogen production, CO_2_ photoreduction, organic pollutant degradation, and bacterial disinfection [40,41,42].

In this paper, we studied the photo-reactivity as a function of specific structural alteration of the phenyl carbon nitride structure, whereas the photoreduction of 4-nitrophenol was studied as a benchmark of the performances.

## 2. Materials and Methods

### 2.1. Materials 

6-phenyl-1,3,5-triazine-2,4-diamine powder (Ph-Triazine, 99%) and H_2_SO_4_ (98%) were purchased from Sigma-Aldrich (St. Louis, MO, USA). Hydrazine Monohydrate (98%) and 4-nitrophenol (98%) were purchased from Alfa Aesar (Haverhill, MA, USA). All the chemicals were used as received without further purification. DI water was used for the preparation of 4-nitrophenol aqueous solution for the photocatalytic tests.

### 2.2. Synthesis of Phenyl-Carbon Nitride

One gram of 6-phenyl-1,3,5-triazine-2,4-diamine powder (Ph-Triazine) was placed in a quartz tube accommodated in a tubular furnace and treated at 400 °C for 1 h. The sample, during the thermal treatment, was covered with a quartz plate to prevent the vaporization and to assure a re-condensation at high temperature. The heating rate was 30 °C/min and the synthesis was performed under constant nitrogen flux (30 mL/min). The produced material was manually grounded to obtain powder-like samples and washed with methanol several times to remove impurities [43]. 

### 2.3. Synthesis of Protonated Phenyl-Carbon Nitride (Hereafter pePhCN)

The pePhCN was prepared by the treatment of PhCN with H_2_SO_4_; 2 g of PhCN powder was added into 40 mL of H_2_SO_4_ (98 wt%) and stirred for 8 h at room temperature. Then, the mixture was slowly poured into 150 mL of deionized water and sonicated for 8 h for exfoliation. After pouring off the clear supernatant, the sediments were rinsed by water again and again. Finally, the pePhCN powder was obtained by drying at 70 °C overnight.

### 2.4. Characterization Techniques

Absorption measurements were obtained by diffuse reflectance spectroscopy utilizing a UV-Vis-NIR Agilent Technologies Cary 5000 (Oxford Instruments, Abingdon, UK). Measurements were performed by using a PbS solid state photodetector using KBr as reference. The reflection configuration measures the diffuse reflection of sample respect to a reference sample which is considered to have a 100% reflectivity. 

Transmission electron microscopy (TEM) (JEOL Ltd., Tokyo, Japan) was performed on a Hitachi H-7000 and on a JEOL JEM 1400 Plus microscope. Finely ground samples were deposited for observation on carbon-coated or on removable formvar silicon monoxide copper grids, both purchased from Ted Pella, Inc. (Redding, CA, USA).

Raman spectra (Iridian Spectral Technologies Ltd., Ottawa, Canada) were acquired in back scattering geometry with excitation wavelength at 1064 nm generated by a Nd:YAG laser, to avoid luminescence contribution in the visible range. The system operates in Stokes region up to 2500 cm^−1^. Measurements were performed in air at room temperature with a spectrometer BWTEK i-Raman EX with a spectral resolution of 9 cm^−1^.

Steady state photoluminescence measurements were performed with a 405 nm laser excitation wavelength, coupled with an optical fiber to an Avantes SensLine AvaSpec-ULS-TEC Spectrometer (Thermo Fisher Scientific, Waltham, MA, USA). The measurements were acquired with 500 ms time window in a 300–800 nm spectral range.

### 2.5. Evaluation of Photocatalytic Activity

The photocatalytic activity of pePhCN was investigated studying the 4-nitrophenol photoreduction using a laser driven Xenon lamp (EQ-99X) (Energetiq Technology, Wilmington, MA, USA) (that present a continuous spectrum in the 200–500 nm spectral range and a constant power density of 1 mW/nm) with a 320 nm filter, as light source.

An amount of 50 mg of catalyst was added to an aqueous solution (50 mL) of 4-NP (5 mg/L) containing 0.02 M hydrazine. Prior to irradiation, the catalyst was stirred in the dark for 60 min to ensure the establishment of adsorption–desorption equilibrium, and the air in reactor was removed by pure N_2_. Every 3 min, 2 mL of the solution was collected and subsequently centrifuged to remove the particles. The trend of the photocatalytic process was analyzed by measuring the maximum absorbance at 400 nm for 4-nitrophenolate ions as a function of the time. 

## 3. Results and Discussion

The protonated and exfoliated phenyl carbon nitride was prepared by delamination of bulk phenyl carbon nitride in sulfuric acid under ultrasonication. Compared to the bulk powder, the exfoliated and protonated product is stable in colloidal suspension, and the nanosheets, with more delicate color, can be collected by evaporating the water [44].

The XRD patterns of the bulk material and pePhCN present two well-defined and relatively broad diffraction peaks at 13.1° and 27.6° related to the interplanar (100) diffraction and the interlayer (002) diffraction of the graphite-like structure of carbon nitride, respectively [43]. The observed peaks in the bulk PhCN indicates that the general 2D structure typical of graphitic-like structures is preserved, but with a smaller distance among the planes (from 3.26 Å to 3.24 Å in g-C_3_N_4_) due to the presence of the phenyl groups within the PhCN structure [45,46].

In the XRD spectrum of pePhCN only a slight variation in the peak at higher angles (lower intensity and position) was observed, which can be attributed to the variation of the interlayer distance after the exfoliation process. 

**Figure 1:**
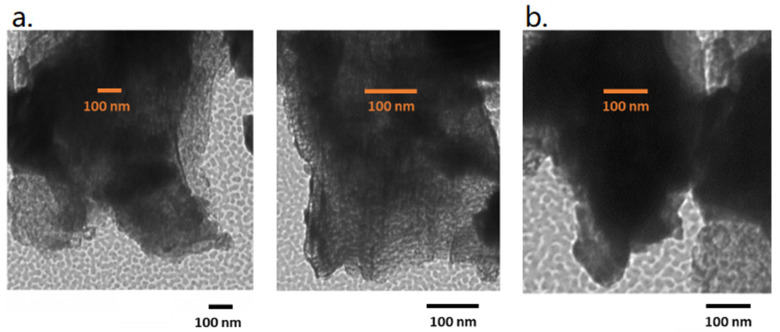
TEM images of pePhCN (**a**) and bulk PhCN (**b**).

The morphology of both systems was studied with TEM measurements (Figure 1). Phenyl carbon nitride shows a typical bulk structure while the modified sample assumes a nanosheet-like aspect. 

In bulk PhCN the values of surface area and pore volume are 3.7 (m^2^·g^−1^) and 0.017 (cm^3^·g^−1^), respectively. In general the protonation and exfoliation process produces ultrathin nanosheets that are characterized by a large surface area and pore volume [47]. Regarding the structural characterization, Raman spectroscopy is classified as a worthwhile technique for the study of the structural and electronic properties in graphitic carbon-based materials [48,49]. To better investigate the consequences of the protonation and exfoliation process in terms of structural, optical and photocatalytic activity, a series of characterizations were carried out studying the differences between PhCN and pePhCN.

**Figure 2:**
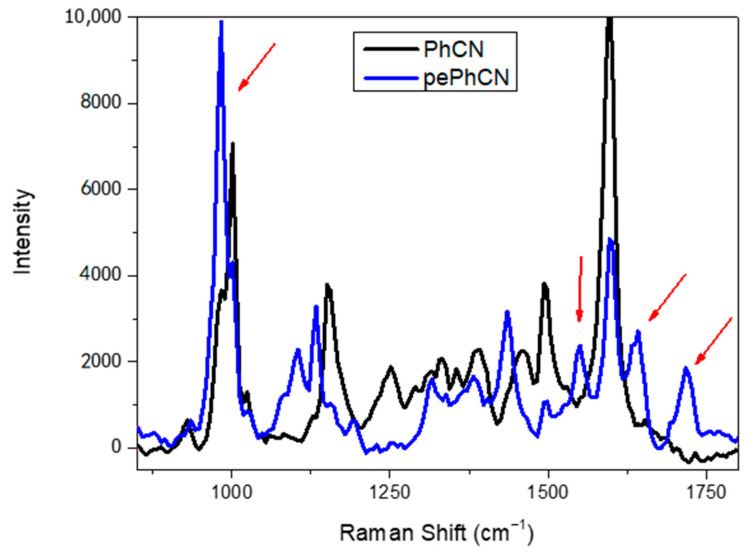
Raman spectra of pure phenyl carbon nitride (black) and protonated and exfoliated phenyl carbon nitride (blue).

Figure 2 shows the Raman spectra of the pure phenyl carbon nitride (black) and the protonated and exfoliated phenyl carbon nitride (blue). 

The spectrum of the phenyl-triazine presents several bands between 900 and 1800 cm^−1^, with prominent peaks at 1601 cm^−1^, related to the motion of the phenyl group, and at 1392 cm^−1^, assigned to conjugated stretching vibration (C–C) between the phenyl ring and the triazine ring [46]. Other prominent figures are at 1000 cm^−1^ (breathing mode) and 674 cm^−1^, related to NH_2_ modes.

**Figure 3:**
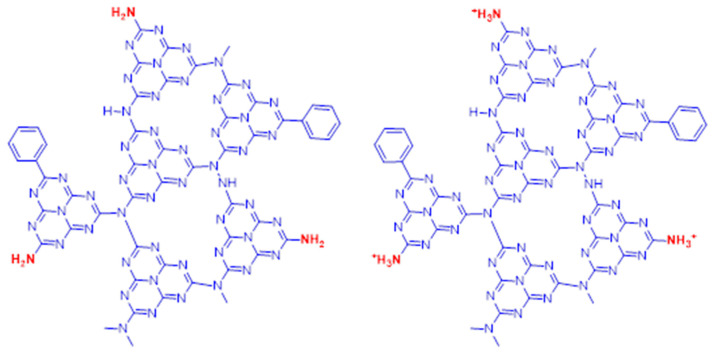
Representation of the protonation process.

The Raman spectrum of the protonated phenyl carbon nitride has the same representative vibrational modes except for some new additional peaks. The peaks at 977 cm^−1^ and 1702 cm^−1^ can be attributed to the symmetric and asymmetric bending of NH_3_^+^ groups, respectively. The antisymmetric bending vibration of the protonated primary amino groups are correlated with the bands around 1630 and 1540 cm^−1^, that are not present in the spectrum of bulk carbon nitride [50,51]. Looking at these results it is possible to state that after the protonation process the skeleton structure does not change but induces the transformation of NH_2_ into NH_3_^+^ terminal groups (Figure 3). Furthermore, the presence of the –SO_3_ species cannot be totally excluded. The Raman signatures of such groups are found in the 950–1100 cm^−1^ region, depending on the specific bonded element. Apart from the already assigned band at 977 cm^−1^, two prominent peaks at 1050 and 1100 cm^−1^ are assignable to the symmetric and antisymmetric stretching of the –SO_3_ modes. However, specific tests should be performed to verify or exclude their presence.

**Figure 4:**
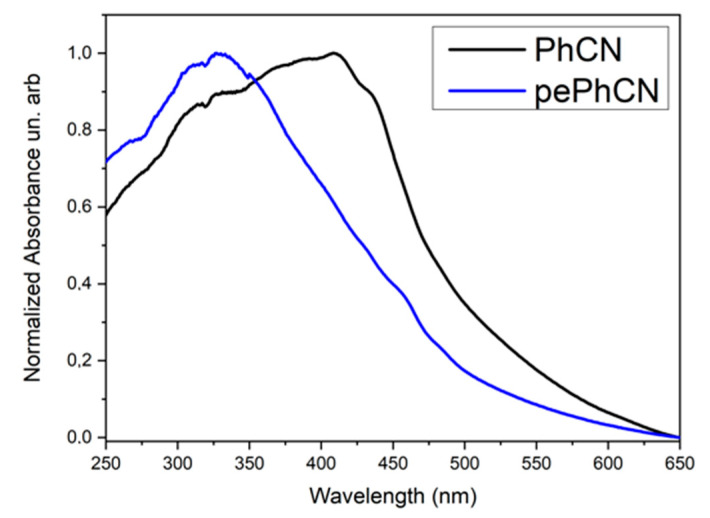
Absorption spectra of PhCN (black) and pePhCN (blue).

Figure 4 shows the absorption profiles of PhCN and pePhCN. Pure phenyl carbon nitride exhibits a broad absorption band that begins at 600 nm with a maximum at 408 nm. This broad absorption is related to the contribution of the π conjugation of phenyl groups as well as the incomplete long range ordering of tri-s-triazine units [52]. The absorption in the protonated and exfoliated phenyl carbon nitride is blue shifted at 340 nm. The blue shift can be attributed to the strong protonation which occurs because of the interaction between the proton (from H_2_SO_4_) and lone pair of electrons of the aromatic nitrogen. Furthermore, it would be connected to the decrease of the conjugation length with a consequent weakening of interaction between the planes, enhancing the contribution of the single layers structure [53]. 

**Figure 5:**
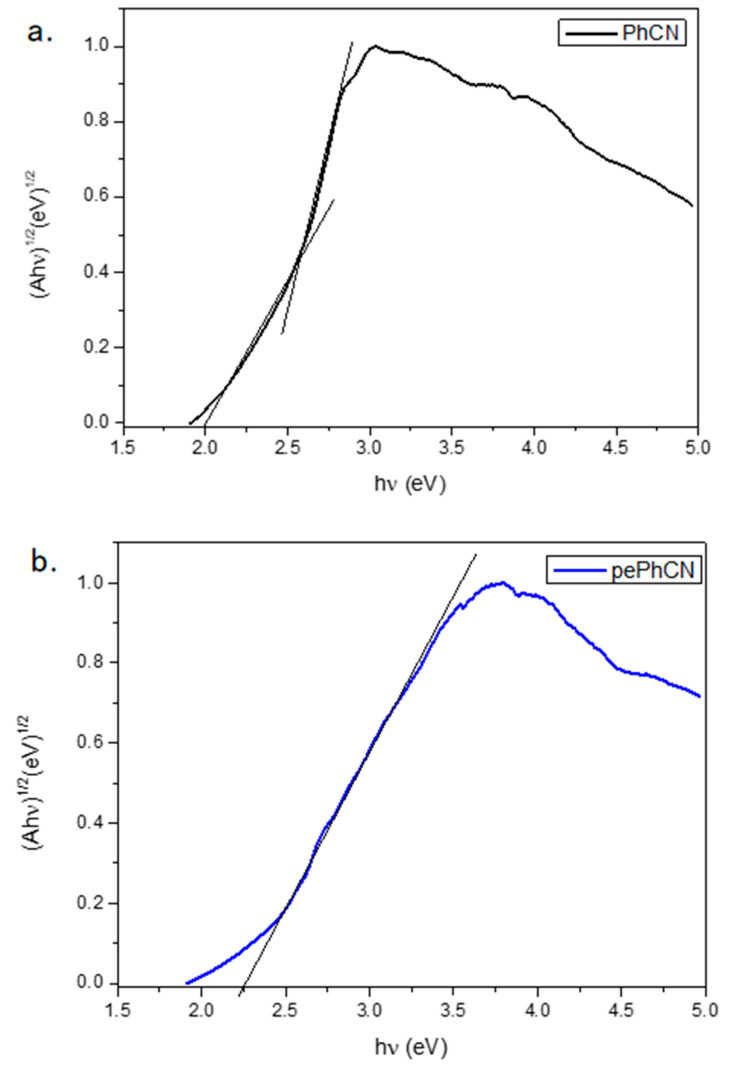
Estimation of the band gap of PhCN (black) (**a**) and pePhCN (blue) (**b**) using the Tauc plot.

The optical band gaps of the CN products were calculated according to the Tauc plots ((αhν)^1/2^ versus hν, in which α is the diffuse absorption coefficient, h is the Planck constant, and ν is the light frequency). As reported in Figure 5 the band gap of the PhCN is estimated at about 2.0 eV (620 nm) and the band gap of pePhCN at about 2.38 eV (521 nm).

In graphitic carbon nitride compounds the band gap strongly depends on the terminal functional groups, if C≡N and C=O narrow down the HOMO–LUMO gap, the O−H could slightly raise the gap. In our PhCN sample, the observation of a multiple band gap could be related to the presence of different functional groups such as phenyl groups [45].

Furthermore, the band gap of carbon nitride compounds is also related to the stacking configuration and, more generally, to the lattice constants in an almost linear relationship. Indeed, the increased interplanar distance in pePhCN samples generates the increase of the band gap, as observed in the absorption measurements [54].

Photoluminescence spectroscopy is considered to be one of the most powerful techniques for characterizing the recombination process of photogenerated excitons.

**Figure 6:**
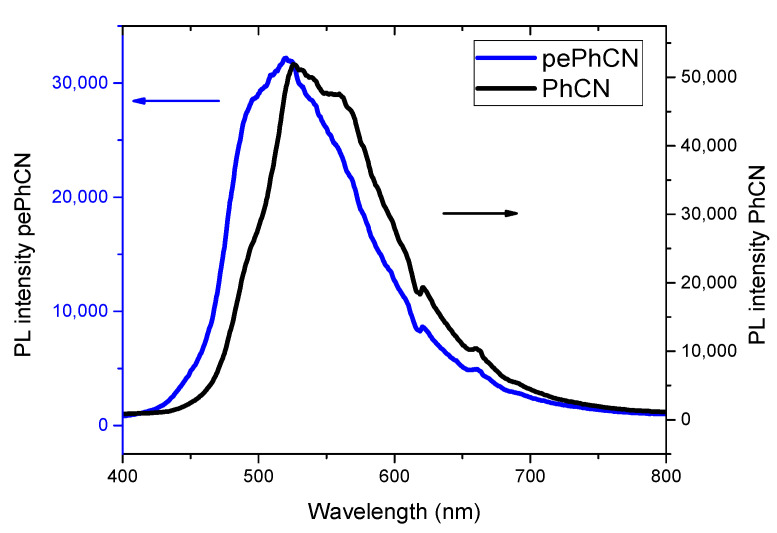
Steady state luminescence spectra of PhCN (black) and pePhCN (blue).

Figure 6 shows the steady state luminescence spectra of PhCN (black) and pePhCN (blue). The exfoliation of layered material is helpful to synthesize more dispersed PhCN which turns out to be conducive to the transfer of photogenerated electron holes and the improvement of the catalytic performance. pePhCN shows a blue shift of the emission peak which is in good agreement with the blue shift observed in the absorption measurements.

The photoluminescence spectra show a remarkably lower intensity for the protonated and exfoliated phenyl carbon nitride, demonstrating that the recombination rate is lower than that of the bulk PhCN [55]. These results suggest that the nanosheets improve the conduction ability of the carriers and reduce the rate of the photogenerated electron–hole pairs [56,57].

The activity of the photocatalyst was tested for the photoreduction of 4-nitrophenol. The absorption peak of the aqueous solution of 4-nitrophenol was centered at 317 nm. After the addition of hydrazine, which reacts with the hydroxylic group of 4-nitrophenol, the absorption peak shifted at 400 nm, indicating the formation of 4-nitrophenolate anion. 

**Figure 7:**
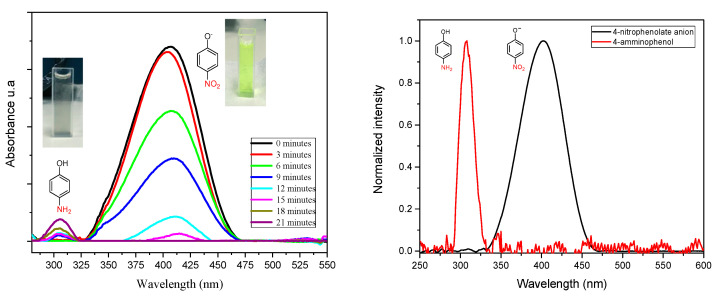
Representation of the conversion of 4-nitrophenolate anion into 4-aminophenol.

In presence of the catalyst and under light irradiation, the intensity of the peak of 4-nitrophenolate anion decreased rapidly and at the main time the formation of a new peak centered at 300 nm, characteristic of 4-aminophenol, was observed (Figure 7).

The photocatalytic activity for the reduction of 4-nitrophenol was evaluated performing different experiments, in different operational conditions. Each experiment was performed three times to test the reproducibility of the process:

(1) pePhCN was used as catalyst in copresence of hydrazine in the aqueous solution under light irradiation.
(2) pePhCN was used as catalyst in copresence of hydrazine in the aqueous solution and the experiment was performed in the dark.
(3) Pure PhCN was used as catalyst in copresence of hydrazine in the aqueous solution under light irradiation.
(4) The experiment was performed without catalyst, with hydrazine and under light irradiation.

**Figure 8:**
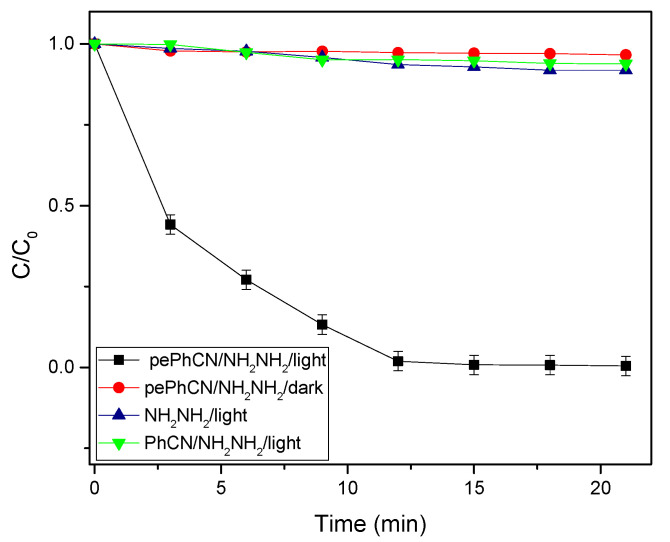
Evaluation of the photocatalytic activity of phenyl carbon nitride and protonated and exfoliated phenyl carbon nitride.

As shown in Figure 8, pure phenyl carbon nitride did not exhibit any photocatalytic activity and no photoreduction happened by increasing the irradiation time. By modifying the surface of the catalyst with the protonation and exfoliation process, the photocatalytic activity was strongly increased and after 15–20 min the total reduction of 4-nitrophenol into 4-aminophenol was obtained. This improvement of the photocatalytic activity is attributed to the facile absorption of the 4-nitrophenolate anion in the positively charged surface of the catalyst. To confirm the crucial role of the catalyst we performed the same experiment in dark conditions with only hydrazine and, in both cases, we did not observe any photoactivity. Furthermore, the experiments were repeated without NH_2_NH_2_ (with light) and no photocatalytic activity was observed for PhCN and pePhCN.

The kinetic characteristic of 4-nitrophenol photoreduction over all the experiments was investigated by using the simplified Langmuir–Hinshelwood model ln(C/C_0_) = kt, where k is the pseudo first order rate constant (min^−1^), C_0_ represents the initial concentration of 4-nitrophenol and C the concentration during the photocatalytic process. The initial concentration was evaluated by performing a calibration curve with five different dilutions of the starting solution of 4-nitrophenol, and then the final concentration for each point of the photocatalytic process was calculated using the Lambert–Beer equation. 

Equations (1)–(4) represent the calculated kinetikc constants of the above mentioned photocatalytic processes. It was possible to highlight a remarkably higher rate constant when the experiment was performed using the protonated and exfoliated phenyl carbon nitride in copresence of hydrazine and light irradiation. The calculated kinetic constants are:

pePhCN/NH_2_NH_2_/light = 2.78 × 10^−1^ min^−1^ (1)

pePhCN/NH_2_NH_2_/dark = 1.95 × 10^−3^ min^−1^ (2)

NH_2_NH_2_/light = 4.57 × 10^−3^ min^−1^ (3)

PhCN/NH_2_NH_2_/light = 3.54 × 10^−3^ min^−1^ (4)

**Figure 9:**
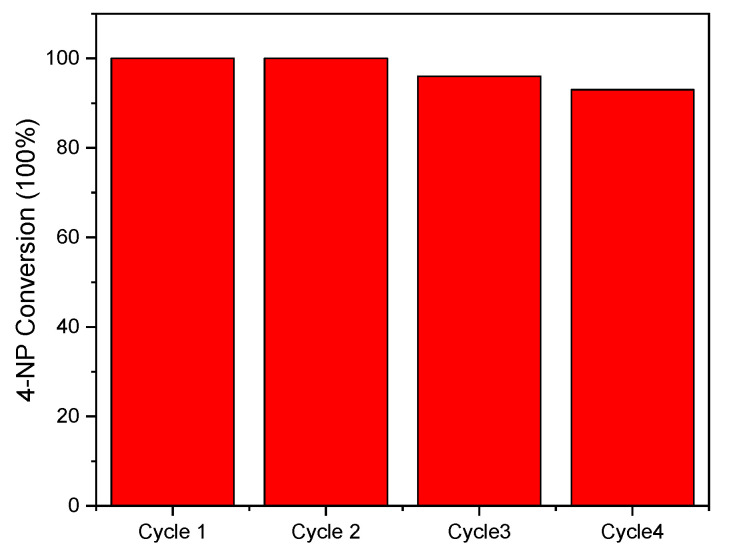
Durability test of pePhCN catalyst.

In addition, the stability of the pePhCN catalyst was evaluated for the photoreduction of 4-nitrophenol performing four consecutive photocatalytic cycles. The removal rate remained almost the same for all the four photocatalytic cycles with a slight decrease for the last one, as shown in Figure 9.

**Figure 10:**
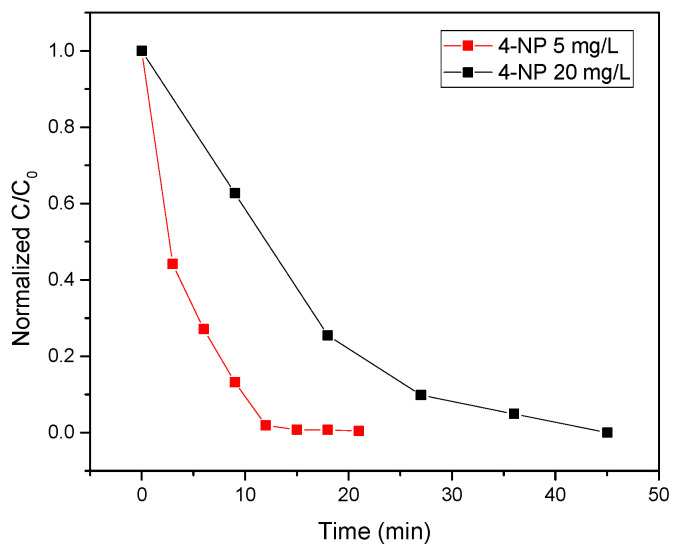
Results of the photocatalytic tests performed using two different concentrations of 4-nitrophenol solution.

Again, tests with different concentrations of 4-nitrophenol were performed. Figure 10 reports the photocatalytic test performed on a wide range of starting concentrations of 4-nitrophenol (5 mg/L and 20 mg/L), indicating a high photocatalytic efficiency in the photoreduction to 4-aminophenol even at the higher concentration.

**Figure 11:**
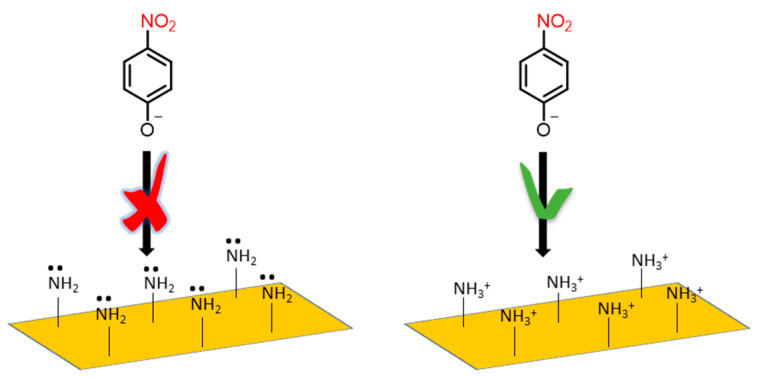
Electrostatic affinity of the 4-nitrophenolate anion with negatively and positively charged surface.

To have efficient photoreduction, the photoelectron–hole pairs should be adequately separated and the transport of the photogenerated electron fast. Hydrazine reacts with 4-nitrophenol producing 4-nitrophenolate anion. In the presence of pure PhCN as a catalyst, the 4-nitrophenolate anion cannot be absorbed on its surface because of the negative charge, and the transfer of photoinduced electrons is restricted. In the case of pePhCN the surface of the catalyst is positively charged, and the 4-nitrophenolate anion can be absorbed successfully allowing the photoreduction reaction (Figure 11). Furthermore, the thin structure of the exfoliated sample decreases the rate of bulk recombination of the photogenerated carriers, increasing the diffusion along the surface.

**Figure 12:**
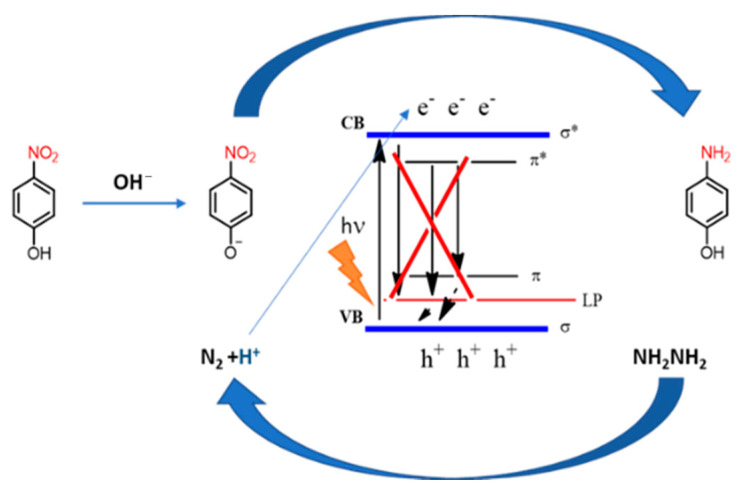
Photocatalytic mechanism.

Looking at the obtained results, in Figure 12 is presented the mechanism proposed for the photoreduction process. Under light irradiation the electrons of the valence band (VB) of the protonated phenyl carbon nitride are excited to its conduction band, living vacancies (h+) on the valence band. The diffusion on the surface of the photogenerated charges is fast and the recombination of the electron–ore pairs is slowed down. These surface holes have a strong oxidative potential that after being captured from the hydrazine can produce N_2_ and H^+^. At the same time, the photoinduced electrons are transferred to the nitro group of 4-nitrophenolate anion and at the end of the process, the amination takes place due to the absorption of H^+^ generated from the oxidation of the hydrazine.

In the PhCN structure with a band gap of 2.0 eV, the LUMO state is positioned at −0.43 eV vs. NHE, while the HOMO level is located at 1.57 eV vs. NHE [9]. Because of the weakening of the interaction between the planes and the enhanced contribution from the single layers structure of the pePhCN, this presents a larger band gap (2.38 eV).

Further analysis and measurements could give more insights into the effective band structure of pePhCN. The exact definition of the potential values for the conduction and valence band will be of great value for the possible development and applications of this new structure. Information on the location of the conduction band edge can be derived by the reduction potential measured by the cyclic voltammetry method, and the zeta potential analysis can give insights into the surface polarity in the different morphologies. Selective measurements in this direction are now ongoing and will be the main topic of future dedicated works.

## 4. Conclusions

In conclusion, the optical and structural properties of a new photocatalyst for the efficient photoreduction process of 4-nitrophenol to 4-aminophenol are reported. The material was obtained by the exfoliation and the protonation of phenyl-doped graphitic carbon nitride. Indeed, despite the absence of photoactivity in the bulk material, a strong effect was found under UV illumination in the modified structure. A schematic model is presented to underline the central role of the positively charged surface obtained in the protonation and exfoliation process. 

Apart from the specific results, this work confirms the crucial role of the surface of the catalysts in the photoreduction reactions, suggesting new strategies for effective pollution remediation.

## Data Availability

The datasets generated during and/or analysed during the current study are available from the corresponding autor on reasonable request.
